# Structure–Property Relationships for n‐Alkylamine and Aliphatic Carboxylic Acid‐Derived Thermomorphic Multiphase Systems

**DOI:** 10.1002/cssc.71019

**Published:** 2026-08-27

**Authors:** Megha Thuruthel Kuriakose, Kaveh Shahbaz, Cameron C. Weber

**Affiliations:** ^1^ School of Chemical Sciences University of Auckland Auckland New Zealand; ^2^ MacDiarmid Institute for Advanced Materials and Nanotechnology Wellington New Zealand; ^3^ College of Science and Engineering James Cook University Townsville Australia

**Keywords:** amines, dye extraction, fatty acids, lower critical solution temperature, phase transition, switchable solvents, thermomorphic multiphase systems

## Abstract

Thermomorphic multiphase systems (TMS) are liquids that display abrupt and reversible changes in miscibility with temperature. TMS based on mixtures of amines and carboxylic acids have been found to exhibit lower critical solution temperature (LCST) behavior, although prior examples have relied on clinical anesthetics as the amine component due to poor understanding of the relationship between the amine structure and LCST behavior. Here, the role of the amine partner of the TMS was systematically investigated, utilizing an array of primary, secondary, and tertiary amines in combination with aliphatic carboxylic acids. Systems featuring tripropylamine (TPA) or dibutylamine (DBA) were uniquely capable of displaying thermoswitchable behavior in the presence of a sufficiently hydrophobic aliphatic carboxylic acid, whereas primary amines and other secondary and tertiary amines investigated did not form TMS. TMS formation could be related to macroscopic hydrophilicity parameters for the amines, providing a foundational basis for future TMS design, and their ability to facilitate extractions was demonstrated. These findings highlight the potential to prepare TMS from scalable, low‐cost amines toward the development of environmentally benign switchable solvent systems.

## Graphical abstract

Systematic investigation of aliphatic amines identified novel thermally switchable solvents and elucidated the physicochemical properties required for their formation. These solvents facilitated efficient and tunable separations, demonstrating that lower critical solution temperature switchable solvents can be prepared from amine‐acid mixtures that are not dependent on complex, environmentally unsustainable amine components.

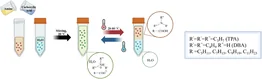

## 1 Introduction

Green chemistry emerged from a desire to reduce the impact of hazards and adverse effects on the environment arising from the chemical industry and chemical products by actively substituting harmful and hazardous substances and processes with safe, environmentally benign alternatives [1]. Solvents are major components of many chemical processes, often used in quantities exceeding all other components, and hence, the development of safer alternative solvents has been a major area of study in green chemistry [2, 3]. A particular focus has been the development of nonvolatile solvents to address the flammability and atmospheric pollution risks posed by conventional volatile organic solvents [4]. However, nonvolatile solvents such as ionic liquids, low‐melting salts, and deep eutectic solvents (DES), low‐melting mixtures, pose challenges with regard to the subsequent recovery of solutes due to their inability to be readily distilled [5]. In addition, distillation of solvents for their removal, even when volatile solvents are used, is energy‐intensive and contravenes the 6th principle of green chemistry, design for energy efficiency [6], highlighting a need to develop alternative processes for the separation and recovery of solutes from solvents.

A proposed solution for these challenges has been the development of solvents that can switch properties such as polarity in response to external stimuli, such as the addition or removal of CO_2_ or changes in pH or temperature [7, 8, 9]. This class of solvents is known as switchable solvents [10, 11]. Some of the earliest examples of switchable systems were CO_2_‐switchable DBU‐alcohol and DBU‐amine systems, which switched between their nonpolar neutral form and their polar ionic form on the addition or removal of CO_2_ [12, 13]. Subsequent development focused on the reversible reaction of water‐immiscible amines such as *N*, *N*‐dimethylcyclohexylamine with carbonated water due to the larger shift in polarity during this switch and water tolerance of these systems; however, many of these CO_2_‐switchable systems are reliant on toxic amines, with attempts to use less hazardous carboxylic acid systems only viable within a very restricted range of conditions where switching can be observed [13, 14, 15]. pH switchable systems work using a similar mechanism, although the addition of stoichiometric quantities of an acid or base triggers the switch [16]. This leads to a rapid switch but generates stoichiometric quantities of salt waste following each transition, which could also affect the long‐term reversibility of the process.

Temperature switchable (thermoswitchable) solvents have a straightforward switching mechanism which relies only on a change in temperature, without the need for any additional substances to be introduced [16]. While temperature changes involve energy input, avoiding the need for energy input corresponding to the enthalpy of vaporization, as is required for distillation, alongside the optimization of transition temperatures for the temperature‐switchable systems, could facilitate these transitions with minimal energy input compared to existing approaches. Thermoswitchable solvents that lead to a change in the number of liquid phases with temperature are known as thermomorphic multiphase systems (TMS) [17, 18, 19, 20]. Traditional TMS involved mixtures of at least two solvents with highly temperature‐dependent miscibility, typically consisting of a highly polar, moderately polar, and/or nonpolar solvent. These systems have been mostly applied to homogeneous transitional metal catalysis as an approach for separation and recovery of the catalyst [18, 19, 20]. More recently, the use of low melting mixtures, often known as DES, as TMS has been investigated, substantially increasing the number of potential TMS available [21].

Two distinct phase separation behaviors can be exhibited by TMS, upper critical solution temperature (UCST) where two (or more) immiscible layers form at lower temperatures and mix at elevated temperatures, or lower critical solution temperature (LCST), where the opposite effect is observed with phases mixing at lower temperatures and separating at elevated temperatures [22]. UCST behavior, which is the more conventional phase separation phenomenon, is the typical behavior of traditional TMS and has also been observed by TMS formed from low melting mixtures composed of alkanolamines or menthol combined with aromatic compounds, often phenol derivatives, or the combination of choline chloride and levulinic acid with aliphatic carboxylic acids [8, 23, 24, 25, 26, 27, 28, 29, 30, 31, 32, 33]. These TMS have been applied to the extraction of organic dyes from water; the extraction of polysaccharides, flavonoids, and phytochemicals from different plant sources; the separation of amino acids in water; and the detection of bisphenols in beverages and pesticides in surface soil [23, 24, 26, 29, 30, 34, 35].

LCST behavior is an unusual phenomenon for liquids that do not feature polymers [36], although some aliphatic amines like triethylamine (TEA) have been reported to exhibit LCST behavior with water [37]. The first low‐melting mixture TMS reported to display LCST behavior with water was comprised of lidocaine and oleic acid with a cloud point (*T*_cp_) of 25 °C, above which two immiscible layers form [34]. The driving force for the LCST behavior was attributed to the acid–base equilibrium between the amine and carboxylic acid components. Subsequent to this discovery, other TMS displaying LCST behavior have been discovered based on mepivacaine and tetracaine [17, 35], although only these clinical anesthetics have been identified as effective partners for LCST TMS behavior.

Toward the generalization and rational design of TMS LCST systems that are not dependent on clinical anesthetics as key components, this work aims to identify structure–property relationships for TMS based on simple aliphatic amines by exploring a series of low melting mixtures composed of primary, secondary, or tertiary amines paired with long‐chain carboxylic acids to determine if they show temperature responsive hydrophilicity. The phase composition and phase diagrams of the identified TMS‐water systems were then studied alongside their ability to extract organic dyes from aqueous solution to understand the relationship between TMS structure and their potential applications as extraction solvents. The aim of this work was to provide a pathway for the development of environmentally benign, scalable TMS solvents.

## 2 Experimental Section

### 2.1 Materials and General Procedures

Chemicals were purchased at the highest purity available and used as received. A complete list of compounds used, chemical suppliers, and purities is available in Table S1. Abbreviations of these compounds used throughout are provided in the following list: triethylamine (TEA), tripropylamine (TPA), tributylamine (TBA), dipropylamine (DPA), dibutylamine (DBA), dipentylamine (DPeA), dihexylamine (DHA), butylamine (BA), pentylamine (PeA), hexylamine (HA), octylamine (OA), hexamethylenediamine (HMA), hexanoic acid (HexA), octanoic acid (OctA), decanoic acid (DecA), dodecanoic acid (DodecA), 2‐ethylhexanoic acid (EHA), methylene blue (MB) and methyl orange (MO).

General procedures were performed within a fumehood as a precaution.

### 2.2 Preparation of Solvent Mixtures

Amine‐acid solvent mixtures were prepared by combining an equimolar ratio of amine and carboxylic acid in a glass vial and stirring at room temperature (~20 °C) for 30 min until a homogeneous liquid formed (Table 1).

**TABLE 1:** Phase behavior observed between 20–95 °C for investigated amine‐acid mixtures. The uncertainty of *T*_cp_ measurements was ±1 °C.

	HexA	OctA	EHA	DecA	DodecA
TEA		M	M	M	
TPA	*T*_cp_ = 85 °C	*T*_cp_ = 79 °C	*T*_cp_ = 28 °C	*T*_cp_ = 69 °C	*T*_cp_ = 59 °C
TBA	I			I	
DPA		M			
DBA	*T*_cp_ = 57 °C	*T*_cp_ = 30 °C	S	*T*_cp_ = 32 °C	*T*_cp_ = 33 °C
DpeA	I	I			
DHA	I	I		S	
BA			M		
PeA			I		
HA		S	I		
OA		S	I		
HMA			S		

*Note* *:* Blank cell = not investigated; I = water immiscible; M = water miscible; S = solid amine–acid mixture; *T*_cp_ = LCST behavior.

### 2.3 Water Miscibility of Solvent Mixtures

Amine‐acid solvent mixtures were combined with an equal mass of water under ambient conditions. These mixtures were stirred (~600 rpm) for 2 min and allowed to settle, and their miscibility was noted. The effect of temperature on these ternary mixtures was monitored visually by gradually heating them in an oil bath from room temperature (~20 °C) up to 95 °C in 10 °C increments, allowing the mixture to stand at the set temperature for approximately 5 min before further increasing if required. If a change in miscibility was observed in the temperature range, the experiment was repeated in that temperature range with an increment of 2 °C, and thermometer reading was noted alongside to identify the exact temperature of transition. Repetition of these experiments identified that *T*_cp_ was reproducible to ±1 °C.

### 2.4 Phase Diagram of TMS

*T*_cp_ values for TMS with varying water contents were studied by adding known quantities of water to a preweighed quantity of the amine‐acid solvent mixture. The *T*_cp_ of the mixture was identified by the method described in Section 2.3. An additional known quantity of water was then added to this mixture, and the process was repeated to establish the phase diagram of selected TMS.

### 2.5 Phase Composition of TMS

The amine‐acid‐water mixtures were heated to the desired temperature above *T*_cp_ to achieve phase separation, and the aqueous and organic layers were separated. Measurements were performed either at 5 °C above *T*_cp_ for each system or at an elevated temperature of around 30 °C above *T*_cp_ (60 °C for all DBA systems and the TPA‐EHA system and 95 °C for the other TPA systems). The systems were left at the specific temperature for 1 h with occasional shaking before separating the layers for further analysis. The composition of amine and carboxylic acid in the aqueous phase was determined by q‐NMR analysis. Approximately equal, but accurately weighed, masses of the aqueous layer and 5% aqueous maleic acid solution were combined and mixed using a vortex mixer, and the ^1^H‐NMR was recorded using a Bruker AVIII400 spectrometer. From reference to the maleic acid peak, the proportion of acid and amine in the aqueous layer was determined. The percentage of water in the organic layer was determined using a Karl–Fischer titrator (TitroLine 7500 KF trace).

### 2.6 pH of the TMS System

pH measurements were obtained using a Mettler Toledo InLab Science Pro‐ISM pH probe connected to a Mettler Toledo SevenCompact S220. The pH meter was previously calibrated with Mettler Toledo technical buffer solution of pH 1.68, 4.01, 7.00, and 10.01, with all measurements obtained at room temperature (~20 °C). pH measurements were obtained for the water used to prepare mixtures, TMS‐water mixtures, and the aqueous phase obtained after phase separation of the TMS‐water mixtures, as described in Section 2.5.

### 2.7 Extraction of Organic Dyes Using TMS

100 ppm solutions of MB and MO were separately prepared in water. Equal volumes of the dye solution and amine–acid mixture were transferred to a vial, mixed, and heated approximately 30 °C above the transition temperature of the TMS for 1 h. The aqueous layer was then isolated, diluted 10‐fold with water, and analyzed using a UV–visible spectrophotometer (Shimadzu UV‐3600 Plus). Neat OctA, TPA, and DBA were also used to extract MB and MO according to the above procedure at temperatures of 95, 95, and 60 °C, respectively. The extraction efficiency was then determined based on Equation (1) below, where *A*_1_ is the absorbance of the extracted aqueous layer and *A*_0_ is the initial absorbance of the dye solution. Calculation of extraction efficiency.

(1) $$\mathrm{EE}=1-\frac{A_{1}}{A_{0}}$$

## 3 Results and Discussion

### 3.1 Amine‐Acid Solvent Mixture Preparation and Thermoswitchable Behavior

A series of primary, secondary, and tertiary amines with systematically varied alkyl chain length were chosen as amine counterparts, and their mixtures with different long‐chain carboxylic acids were prepared as noted in Table 1. Most of the resultant amine‐acid mixtures were moderate to highly viscous liquids at room temperature; however, a few secondary amine systems (DBA‐EHA and DHA‐DecA) and many of the primary amine systems, such as HA and OA and the diamino HMA, formed solids (Table 1, highlighted orange) when mixed with the fatty acids. For the primary amines, the use of EHA, a branched fatty acid, allowed the formation of a liquid mixture under ambient conditions. Surprisingly, EHA formed a solid when combined with DBA despite DBA forming homogeneous liquids with all other acids tested, although the origin of this behavior was not further explored. Only the mixtures which were liquid under ambient conditions were further studied for their temperature‐responsive hydrophilicity.

The determination of the temperature‐dependent behavior of the amine‐acid‐water mixtures was performed at a 1:1 mass ratio of the combined amine and acid: water. The mixtures were gradually heated on an oil bath from room temperature to 95 °C in 10 °C increments, with each temperature held for approximately 5 min. If phase separation was observed, a more accurate *T*_cp_ estimate was obtained by slowly heating around the observed transition temperature, with the internal temperature of the solution determined via a thermometer and the temperature where the onset of turbidity was observed noted as *T*_cp_. Temperatures above 95 °C were not investigated owing to the proximity to the boiling point of water.

For the tertiary amines, it was found that all TEA mixtures were miscible with water, and all TBA mixtures were immiscible across the temperature range explored. On the contrary, TPA formed TMS when combined with HexA, OctA, EHA, DecA, and DodecA. TPA‐HexA formed a very thin upper layer and translucent lower layer at ~85 °C, suggesting that this system lies at the borderline of miscibility. For this reason, it was not taken forward for further investigations. For the secondary amines tested, DPA formed water‐miscible mixtures, and DPeA formed water‐immiscible mixtures over the investigated range of temperatures. However, DBA formed TMS with HexA, OctA, DecA, and DodecA, with DBA‐EHA systems forming a solid as noted earlier, so they were not investigated. While DBA‐HexA did lead to phase separation, the phases formed were very unequal in volume, with a much smaller upper organic layer compared to the larger lower aqueous layer, showing similar miscibility behavior to TPA‐HexA. None of the primary amines explored in this study exhibited temperature switchable hydrophilicity. Solids were formed by the combination of OA‐OctA, HA‐OctA, and HMA‐OctA. The liquid mixtures formed by the combination of PeA‐EHA, HA‐EHA, and OA‐EHA were water immiscible, and BA‐EHA was water miscible at all investigated temperatures (Table 1), indicating that no alkyl‐chain‐dependent miscibility could be determined for the primary amines with this carboxylic acid component. Note that no UCST behavior was seen for any of the systems investigated.

**FIGURE 1:**
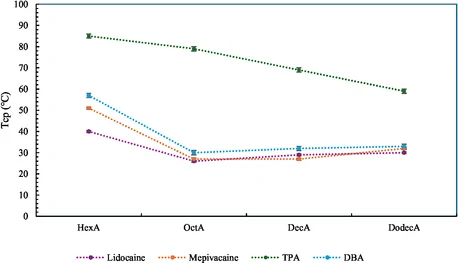
Comparison of *T*_cp_ values for TMS based on TPA and DBA (this work) with published lidocaine and mepivacaine values [17].

An interesting trend that can be observed in Table 1 is that TPA‐containing TMS display changes in *T*_cp_ with changes in the alkyl chain length and structure of the acid partner, whereas, with the exception of HexA, DBA does not. Figure 1 depicts the reported *T*_cp_ values for the TMS studied here as well as those reported for the lidocaine and mepivacaine systems in the literature [17]. Comparison of these values indicates that the general trend in DBA *T*_cp_ values was consistent with those of lidocaine and mepivacaine, although the DBA‐acid systems showed slightly higher absolute *T*_cp_ values for the same acid partner (Figure 1). TPA, on the other hand, clearly demonstrates an approximately linear decrease in *T*_cp_ with increasing alkyl chain length of the carboxylic acid partner from 6–12.

These trends suggest that the LCST behavior in terms of *T*_cp_ changes from being driven by a combination of the hydrophobicity of the acid and the acid–base equilibrium, such as in the case of TPA and TMS featuring HexA, to being exclusively driven by the acid–base equilibrium, which results in *T*_cp_ values that are independent of the carboxylic acid alkyl chain length. These trends suggest that the origin of the LCST behavior therefore likely arises from a combination of the hydrophobicity of the TMS components and their inherent acidity/basicity.

### 3.2 Correlation of Thermoswitchable Behavior With Physical Properties of TMS Components

Given the link between amine selection and the formation of TMS systems, the relationship between the physicochemical properties of the amines that formed TMS and those that did not was explored. The octanol‐water partition coefficient (log P)of the amines and the pKa of their corresponding ammonium salts were obtained from the literature to identify if any relationships between these parameters could be observed for these systems (see Table S2).

From Table S2, it is evident that the pKa values did not vary substantially across the series of amines investigated, including amines that did and did not yield TMS systems. This indicates that, at least for these systems, the basicity of the amine was not a determining factor. It should be noted, though, that the ammonium salts of these amines possessed pKa values 2–3 units greater than those of the other amines that have been explored as TMS (lidocaine, mepivacaine, and tetracaine), highlighting that using more basic amines does not prohibit the formation of TMS.

With respect to log P, the amines that formed TMS (TPA, DBA, lidocaine, mepivacaine, and tetracaine) possess log P values that range from 1.95 to 3.6. This is in contrast with the amines that formed mixtures that were miscible with water at all temperatures (TEA, DPA, and BA), which uniformly possessed log P values lower than 2. Secondary and tertiary amines that formed mixtures that were immiscible with water at all temperatures (TBA and DPeA) possessed log P values higher than 3.5, highlighting that, at least for secondary and tertiary systems, a log P within the range of 1.95–3.6 appears necessary to form TMS. The exceptions to this were the primary amines PeA, HA, and OA that possess log P values from 1.49 to 3.1 but uniformly displayed water‐immiscible behavior with EHA despite their low log P. This indicates that primary amines display reduced water miscibility in the presence of the carboxylic acid additive, although the specific origin of this remains unclear.

It might be conceived that the significantly lower *T*_cp_ values of DBA compared to the equivalent acid mixtures with TPA may be due to a difference in the hydrophobicity of the two systems. However, both amines possess similar log P values, which suggests that the origin of this behavior is not due to the inherent hydrophobicity of the amines. An alternative explanation is that this discrepancy is due to different impacts of temperature on the basicity of DBA and TPA, noting that such differences have been seen between secondary and tertiary amines previously [38]. Given the quite substantial differences in *T*_cp_ observed and the proximity to 25 °C for the DBA systems, where the pKa's of the conjugate acids used for comparison were obtained, it would seem unlikely that this alone could account for this difference.

Instead, it is most likely that the change in *T*_cp_ behavior between DBA and TPA systems arises from the difference in specific intermolecular interactions from synergistic effects between the mixture constituents. For example, it could be envisaged that DBA/acid mixtures can form extended hydrogen‐bonding networks due to the available N‐H of the amine interacting with nearby carboxylic acid headgroups. This is an interaction inaccessible to the TPA tertiary amine system and could lead to more consistent and lower‐temperature separation of amine/acid components in the case of DBA/acid mixtures.

### 3.3 Phase Composition of TMS

An important factor in the use of TMS is the extent to which the organic components leach into the aqueous phase, as this affects the potential of these liquids to be recycled and can lead to contamination of compounds isolated in the aqueous phase. The concentrations of the organic components (amines and carboxylic acid) in the aqueous and organic phases were monitored using q‐NMR and a maleic acid standard. The water content in the organic layer was determined by Karl‐Fischer titration. The composition of these phases was monitored at ~5 °C above *T*_cp_ (shown as T_cp_) and approximately 30 °C above *T*_cp_ (high temp, Table 2) to investigate the compositional changes.

**TABLE 2:** Phase composition of the aqueous and organic phases after phase separation.

TMS	Percentage of organics in aqueous phase				Percentage of water in organic phase
	Wt% amine		Wt% acid		
	*T*_cp_	High temp	*T*_cp_	High temp	High temp
TPA‐OctA	4.7 ± 0.94	2.8 ± 0.53	5.1 ± 1.3	2.8 ± 0.58	6.9 ± 0.39
TPA‐DecA	2.7 ± 1.5	0.69 ± 0.10	2.1 ± 1.1	0.74 ± 0.08	4.6 ± 0.77
TPA‐DodecA	2.4 ± 1.2	0.37 ± 0.01	3.5 ± 1.5	0.39 ± 0.02	3.5 ± 0.19
TPA‐EHA	5.0 ± 1.7	5.4 ± 0.69	4.9 ± 1.7	4.9 ± 0.75	3.3 ± 0.16
DBA‐OctA	1.6 ± 0.23	1.9 ± 0.13	1.8 ± 0.18	1.9 ± 0.17	21 ± 2.6
DBA‐DecA	0.88 ± 0.28	0.68 ± 0.19	0.97 ± 0.45	0.71 ± 0.19	16 ± 1.7
DBA‐DodecA	2.0 ± 0.78	0.72 ± 0.02	2.9 ± 1.0	0.97 ± 0.12	16 ± 2.1

*Note:* High temp = 60 °C for all DBA systems and TPA‐EHA and 95 °C for all the other TPA systems. *T*_cp _= Temperature held at 5 ± 1 °C above the cloud point of each of the TMS systems.

At *T*_cp_, it was found that the proportion of amine and carboxylic acid in the aqueous phase was higher in the TPA systems compared to the DBA systems. The highest amount of amine and acid in the aqueous phase was in TPA‐OctA system (4.7% amine and 5.1% acid). Higher temperature conditions (high temp, Table 2) led to a reduction in organic components in the aqueous phase. The exception to this general trend was for TPA‐EHA system, which showed no significant change in separation at higher temperatures.

The water content in the organic phase of TPA systems decreased with the increasing hydrophobicity of the acid on going from OctA to DodecA (6.9%–3.5%). For the DBA systems, the water content in the organic phase was highest for DBA‐OctA (21%), while the DecA and DodecA systems showed lower values (16% in both cases). This suggests that the organic phase accommodates a lower water content as the hydrophobicity of the acid increases while also highlighting that all DBA mixtures investigated are more hydrophilic than the TPA mixtures.

The literature values of lidocaine‐DecA indicated that with an increase in temperature, the water content in the organic phase reduced; however, the percentage of acid and amine in the aqueous phase remained unchanged [17]. In that study, it was noted that there was a general improvement in the purity of both the aqueous and organic phases with an increase in temperature. In the lidocaine‐DecA systems, it was found that under the higher temperature conditions, the aqueous phase contained 0.4% lidocaine and 0.7% DecA in the aqueous phase, and the water content in organic phase was 12%. This indicated that the systems in our investigation have comparable separation efficiency to the previously reported lidocaine‐DecA system.

### 3.4 pH of the TMS System

The pH of the TMS‐water systems was studied upon mixing of the amine and acid under ambient conditions and of the aqueous phase after phase separation at a temperature ~30 °C above the *T*_cp_ for each TMS–water mixture, with results reported in Table 3. The water used was found to be slightly acidic (pH = 5.34) in the absence of any acid or amine solutes, likely due to dissolution of CO_2_ in the deionized water.

**TABLE 3:** pH of the TMS mixture and aqueous layer after separation. Reported errors are standard deviations of replicate experiments.

TMS	pH of TMS mixture	pH of aqueous layer
TPA‐OctA	8.09 ± 0.15	6.87 ± 0.01
TPA‐DecA	8.23 ± 0.16	7.85 ± 0.01
TPA‐DodecA	8.17 ± 0.19	8.70 ± 0.01
TPA‐EHA	8.26 ± 0.01	7.08 ± 0.01
DBA‐OctA	8.50 ± 0.05	8.55 ± 0.19
DBA‐DecA	8.71 ± 0.02	8.99 ± 0.11
DBA‐DodecA	8.67 ± 0.23	9.19 ± 0.02
Water	5.34 ± 0.05	

For the TMS mixtures before separation, the pH of the DBA systems was slightly higher than that of the TPA systems, consistent with the higher reported pKa for DBA (ESI). Both systems showed a higher pH than that of the water used. The pH of the aqueous layers after separation for both DBA and TPA systems was also higher relative to the pH of water. Based on the phase compositions depicted in Table 2, there was a slightly higher quantity of amine relative to acid on a mole basis in the aqueous phase of all systems. This could account for the higher pH value of the aqueous layer relative to the water used. There is also a clear increase in the pH of the aqueous layer as the alkyl chain length of the acid increase. While this could be hypothesized to result from reduced acid concentration in the aqueous phase, the results in Table 2 demonstrate that such a decrease occurs in conjunction with a decrease in the concentration of amine, with the ratios of these compounds not systematically changing with alkyl chain of the acid. Hence, the precise origin of the pH changes with acid alkyl chain length remains unclear.

### 3.5 Effect of Water Concentration on Phase Behavior

**FIGURE 2:**
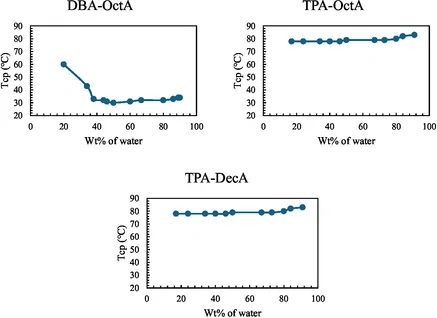
*T*_cp_ of DBA‐OctA, TPA‐OctA, and TPA‐DecA systems with varying water content. Uncertainty of *T*_cp_ measurement was ±1 °C and was not included for clarity.

The impact of water content on the observed *T*_cp_ was determined for DBA‐OctA, TPA‐OctA, and TPA‐DecA (Figures 2 and S1) to gain information about the phase diagrams of these mixtures. It is evident from the phase diagram of DBA‐OctA (Figure 2) that *T*_cp_ decreased from 60 to 34 °C with increasing water content from 20 to 40 wt% water, with *T*_cp_ varying only slightly from 30–34 °C as the water content was further increased. Both TPA‐OctA and TPA‐DecA showed only small variation in *T*_cp_ (Δ*T*_cp_ < 10 °C) as the water content increased from 20 to 90 wt%, although both displayed a slight increase in *T*_cp_ across this range. Previously reported lidocaine‐oleic acid (1:1) systems have also been found to display relatively small changes in *T*_cp_ (Δ*T*_cp_ < 10 °C) with varying water content (10–90 wt%), with increases only observed toward the highest and lowest water content, consistent with the tertiary amine system explored here [29]. However, in tetracaine‐DodecA (1:1), a much more pronounced impact of water has been observed, with *T*_cp_ varying from 15–70 °C as the water content was varied from 20 to 100 wt%, with an initial decrease in *T*_cp_ from 70–15 °C as the water content increased from 20 to 50 wt%, followed by a more gradual increase from 15–39 °C as the water is further increased from 50 to 100 wt% [35]. This illustrates that the phase behavior of TMS can be very system‐specific and likely arises from a balance of hydrogen‐bonding interactions and solvophobic exclusion of constituents in these mixtures.

### 3.6 Dye Extraction by TMS

**FIGURE 3:**
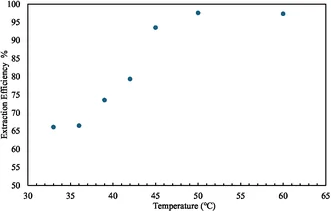
MB extraction efficiency of DBA‐DodecA with variation in temperature.

**FIGURE 4:**
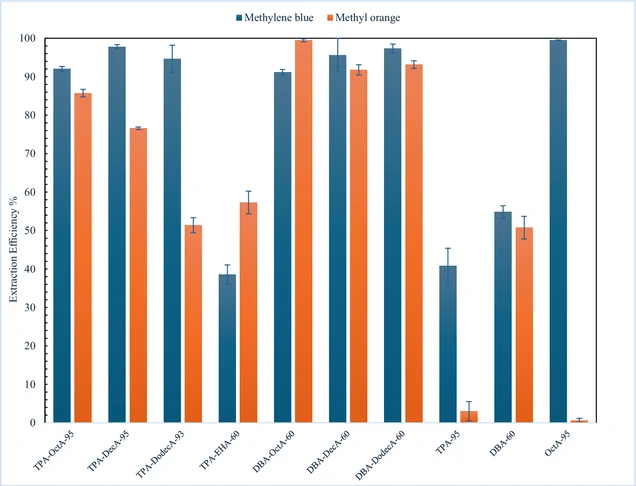
Dye extraction efficiencies of TMS systems and their liquid component. *X*‐axis labels depict both the TMS/component and the temperature of the extraction performed.

The ability of the different TMS as well as neat TPA, DBA, and OctA to act as extraction solvents was studied using MB and MO as cationic and anionic dye extraction models, respectively. The extraction efficiencies (EE) were calculated from UV–visible absorbance data according to Equation (1). Before screening a range of TMS, the effect of temperature was explored for a representative TMS (DBA‐DodecA). The EE was found to increase with temperature up to approximately ~30 °C above the *T*_cp_ of the system (Figure 3). Given this temperature effect, all other dye extractions were performed 30 °C above *T*_cp_ or at 95 °C, whichever was lower. These extractions were performed for the TMS as well as neat TPA, DBA and OctA and are depicted in Figure 4.

From Figure 4, it is clear that MB was successfully extracted with EE > 90% for all TMS, apart from TPA‐EHA, which had an EE of 39%. To assess whether this extraction efficiency could be attributed to individual components of the mixture rather than the mixture as a whole, TPA, DBA, and OctA (1:1 volume ratio) were investigated as extraction solvents, as these are inherently liquid constituents of these mixtures. TPA and DBA without the acid component yielded moderate EE (40%–50%) of MB, indicating that while the amine component can aid extraction, the EE is reduced. However, neat OctA was also able to extract MB with EE > 90%, indicating that the extraction of MB may be linked primarily to the organic acid component, likely through dispersive interactions with the long alkyl chain of the acid in conjunction with hydrogen‐bonding interactions with the carboxylic acid headgroup [39, 40]. This accounts for the lower EE for the EHA‐containing mixture which possesses a shorter and more hindered alkyl chain, reducing available dispersive interactions.

Greater variation was observed for the anionic MO. MO was extracted at >90% EE for all DBA‐acid systems; however, a decrease in EE from 85% to 51% could be observed with increasing alkyl chain length for the TPA‐derived TMS (DodecA to OctA). Investigation of the liquid constituents of these mixtures identified that DBA without the acid component led to moderate EE (50%), whereas TPA and OctA led to very low levels of extraction (EE of 3% and 0.6%, respectively). These outcomes can be rationalized by consideration of hydrogen‐bonding interactions in the organic phase of the two classes of TMS. For the DBA system, hydrogen bonds formed between the acid and amine would help increase the strength of the DBA N─H hydrogen bond donor group, which would be available to interact with the anionic sulfonate group on MO, aiding its solvation in the organic phase. For the TPA‐based systems, hydrogen‐bonding interactions between the acid and amine would compete with the acid‐MO hydrogen bonding interactions, meaning that the solvation of the polar MO would be more limited, with its solubility decreasing as the hydrophobicity of the acid increases. The synergistic nature of this interaction is supported by the EE of the individual constituents, which demonstrates that DBA, TPA, and OctA individually were all less effective at extracting MO than their corresponding mixtures, substantially so in the case of TPA and OctA, emphasizing the synergistic effect of the interaction. These results demonstrate that not only can these TMS be used as extraction solvents but that the molecular interactions within these solvents can be tuned to affect the solubility of solutes, and the use of these mixtures can lead to synergistic effects beyond that achievable by individual constituents of the mixture, as demonstrated by the variable EE of the MO dye.

Lidocaine‐DecA has been reported to yield an EE of 99% for the MO dye. This can be attributed to the functionality present in the lidocaine molecule, as the amide group in the amine may facilitate hydrogen‐bonding interactions with the anionic MO, and the aromatic groups may also engage in π–π type interactions, which could enhance the solubility of this dye molecule. This reiterates the potential tunability of these systems toward achieving the desired separation [17].

### 3.7 Green Chemistry Considerations

Green chemistry considers all aspects of a chemical product or process, including its preparation, use, and end of life. An important consideration in green solvent design is the synthetic pathway of the solvent as this determines the inherent environmental impact of its production. A simple tool for evaluating this involves consideration of the synthesis tree, which outlines all steps currently used industrially for a given compound [5, 41]. Synthesis trees of TPA, DBA, lidocaine, and tetracaine are provided in the supporting information (Figures S2–S5). These trees illustrate that lidocaine requires 31 steps and tetracaine 28 steps to produce, compared to only 8 steps required for both TPA and DBA. This illustrates that the synthesis of TPA and DBA will inherently produce less waste due to the reduced number of synthetic and purification steps. Notably, the preparation of lidocaine also requires several highly hazardous reagents and reaction steps, including thionyl chloride and the need for chlorine due to the presence of chlorinated compounds during the synthetic pathway.

An additional benefit of the substantially reduced synthetic footprint is the much lower inherent cost of TPA and DBA compared to lidocaine and tetracaine, enhancing the economic viability of these solvents. However, the toxicity and safety data [35, 36, 37, 38] suggest that aliphatic amines are more toxic and corrosive than lidocaine and tetracaine, indicating that further analysis is required to establish the more benign alternative. To help systematically evaluate the relative environmental and human health impacts of these solvent constituents, the criteria proposed under the CHEM21 solvent selection guide were applied [42], with the evaluation for the amines and acids used in this work as well as lidocaine, oleic acid, and tetracaine provided in Table 3. The CHEM21 selection guide evaluates solvents on the basis of easily accessible physical properties and toxicological/eco‐toxicological data commonly found in Safety Data Sheets. Each of the categories is evaluated as a score, with higher values representing higher levels of hazard. As is evident from Table 4, TPA and DBA possess reduced environmental scores relative to lidocaine but higher levels of hazard in terms of health and safety, with DBA being more hazardous than TPA. This indicates that while DBA alternatives should be avoided from a sustainability perspective, TPA‐based TMS systems present similar categorization with regard to solvent desirability as lidocaine and tetracaine, with substantially reduced embedded cost and environmental waste generation from its synthesis. As all constituents are considered problematic from the solvent selection guide perspective, it is apparent that further work is still required in order to identify more environmentally sustainable LCST TMSs; however, this work has demonstrated that such systems can be derived from structurally simple, easy‐to‐prepare amines rather than more synthetically complex compounds [42].

**TABLE 4:** Solvent selection guide ranking of TMS constituents based on CHEM21 criteria [42].

Chemical	Safety score	Health score	Environment score	Ranking
TPA	3	7	5	Problematic
DBA	3	9	5	Hazardous
Lidocaine	1	6	7	Problematic
Tetracaine	1	6	7	Problematic
OctA	1	7	7	Hazardous
DecA	1	2	7	Problematic
EHA	1	9	7	Hazardous
DodecA	1	4	7	Problematic
Oleic A	1	1	7	Problematic

*Note:* Shading represents the traffic light color‐coding system introduced for these criteria.

## 4 Conclusions

This study identified new TMS systems composed of simple tertiary and secondary amines (TPA and DBA), demonstrating that a wider range of amines can be used for the preparation of TMS exhibiting LCST behavior than those that have been reported to date. The formation of TMS appears to arise primarily from the hydrophobicity of the amine and acids, with a log P of approximately 2–3 appearing to be a necessary condition for secondary and tertiary amines to form TMS when accompanied by sufficiently hydrophobic (log P > 3) acids. The basicity of the amine does not appear to be a critical factor, at least as long as the pKa of its conjugate ammonium salt in water lies in the range of 7–11. From the phase diagrams of the investigated systems, it is evident that the water content causes a significant variation in *T*_cp_ for the secondary amine (DBA) systems; however, the tertiary amine (TPA) systems exhibit only minor changes in *T*_cp_, despite substantial changes in the water content. It is also evident that the temperature of the separation is an important factor, as increasing the temperature beyond *T*_cp_ reduced the level of contamination of organic components in the aqueous phase and improved the extraction efficiency of model solutes. The use of the branched acid EHA resulted in unusual outcomes compared to the linear acids, reducing the extraction efficiency of the TMS and highlighting the need to further investigate the impact of chain branching on the properties of these solvent systems. The use of synthesis trees revealed that TPA and DBA likely have substantially reduced cost and environmental impact from their syntheses due to the absence of highly hazardous synthetic steps and the markedly reduced number of steps required compared to lidocaine and tetracaine. However, implementation of CHEM21 solvent selection criteria highlighted that despite the reduced embedded environmental hazard, DBA and TPA both present higher risks with regard to health criteria than lidocaine and tetracaine, highlighting the need for the development of new LCST TMS based on our enhanced understanding of the properties required for these systems to form.

## Funding

This study was supported by MacDiarmid Institute for Advanced Materials and Nanotechnology.

## Conflicts of Interest

The authors declare no conflicts of interest.

## Supporting information

Supplementary Material

## Data Availability

The data that supports the findings of this study are available in the supporting information of this article.
