# Supplementary material for: Structure–Property Relationships for n‐Alkylamine and Aliphatic Carboxylic Acid‐Derived Thermomorphic Multiphase Systems
Source: ChemSusChem. 2026 Aug 27;19(17):e71019. doi: 10.1002/cssc.71019 (PMC13520806; doi:10.1002/cssc.71019)
Supplement: Supplementary file 1 [file CSSC-19-e71019-s001.pdf]

Supplementary material of

Structure-Property Relationships for n-Alkylamine and Aliphatic Carboxylic Acid-Derived Thermomorphic Multiphase Systems

Megha Thuruthel Kuriakose[a, b], Kaveh Shahbaz[c], Cameron C. Weber[a, b]*

[a] School of Chemical Sciences, University of Auckland, Auckland, New Zealand

[b] MacDiarmid Institute for Advanced Materials and Nanotechnology, Wellington, New Zealand

[c] College of Science and Engineering, James Cook University, Townsville, Australia

Email: cameron.weber@auckland.ac.nz

Table S1: Chemicals used, suppliers and purity

Chemical	Supplier	Purity
Triethylamine (TEA)	JT Baker	≥99%
Tripropylamine (TPA)	Sigma-Aldrich	≥98%
Tributylamine (TBA)	Janssen	99%
Dipropylamine (DPA)	Sigma-Aldrich	99%
Dibutylamine (DBA)	AK Scientific. Inc	>99%
Diamylamine (DAA)	AK Scientific. Inc	98%
Dihexylamine (DHA)	AK Scientific. Inc	98%
Butylamine (BA)	ECP, Ltd	99%
Amylamine (AA)	AK Scientific. Inc	98%
Hexylamine (HA	Oakwood chemical	98%
Octylamine (OA)	Sigma-Aldrich	99%
Hexamethylenediamine (HMA)	Sigma-Aldrich	98%
Hexanoic acid (HexA)	Sigma-Aldrich	≥98%
Octanoic acid (OctA)	Acros Organics BVBA	99%
Decanoic acid (DecA)	Sigma-Aldrich	≥99.5%
Dodecanoic acid (DodecA)	ECP,Ltd	99%
2-Ethylhexanoic acid (EHA)	AK Scientific. Inc	95%
Maleic acid	BDH	99%
Methyl orange (MO)	Ajax	NA
Methylene blue (MB)	Riedel-de Haën	NA

Table S2: Literature reported pKa and Log P values of the amines and carboxylic acids used

Amine	Log P	pKa
TEA[1]	1.45	10.76
TPA [1]	2.79	10.46
TBA[2]	4.46	10.89
DPA [1]	1.67	10.93
DBA [1]	2.83	11.25
DPeA [2]	3.76 (est)	11.16
DHA	4.5	11.01
BA [1]	0.74	10.56
PeA [1]	1.49	10.61
HA [1]	2.06	10.60
OA [1]	3.09	10.61
HMA[2]	0.35 (est)	10.7
Lidocaine[3]	2.48	8.19
Mepivacaine [3]	1.95	7.92
Tetracaine [3]	3.56	8.59
HexA [1]	1.92	4.87
OctA [1]	3.05	4.89
EHA [1]	2.64	4.8(est)
DecA [1]	4.09	4.9(est)
DodecA [1]	4.6	5.3

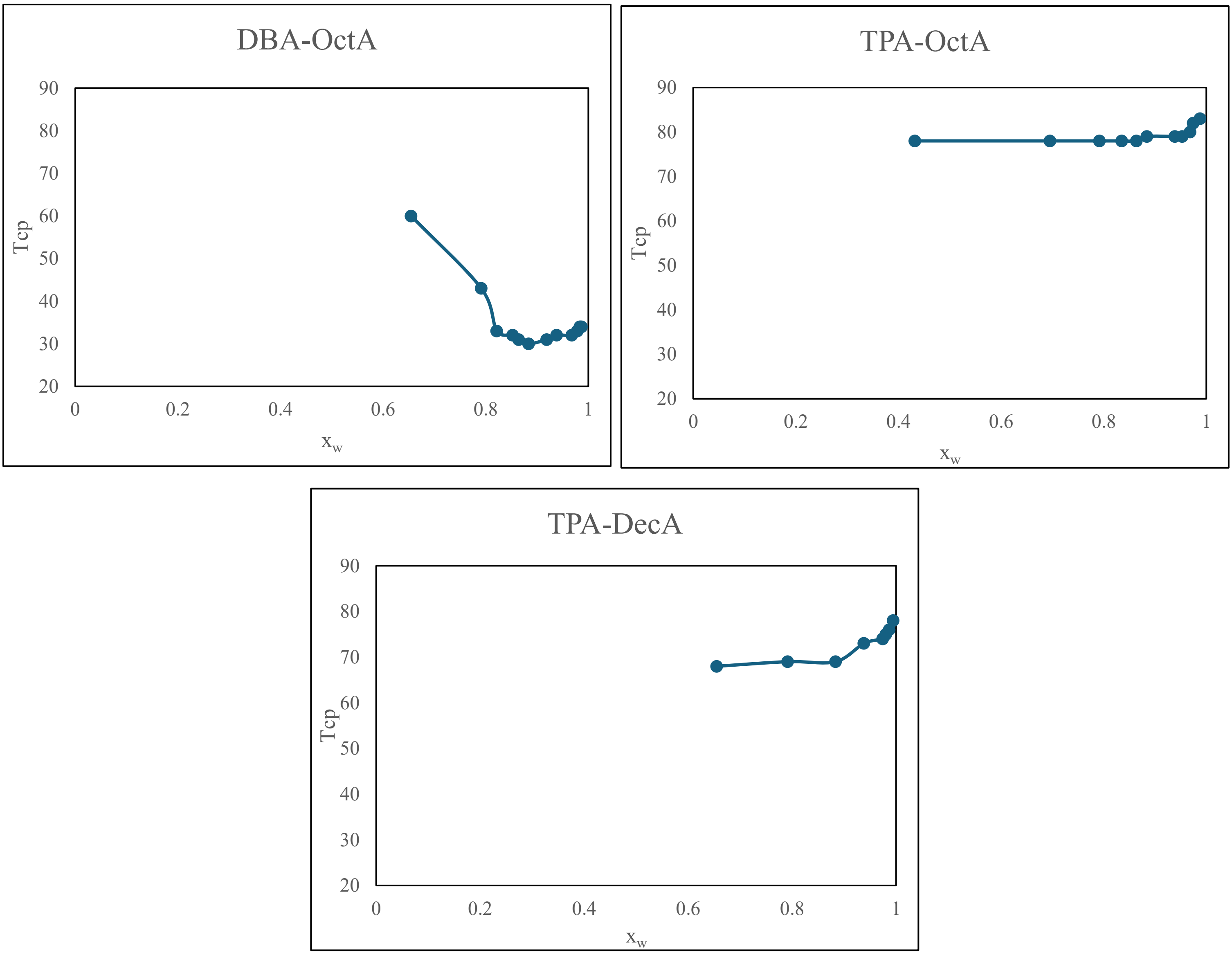

Figure S1: Phase diagram of DBA-OctA, TPA-OctA and TPA-DecA systems with varying mole fractions of water. Uncertainty of T_{cp} measurements (±1 °C) are not shown for clarity.

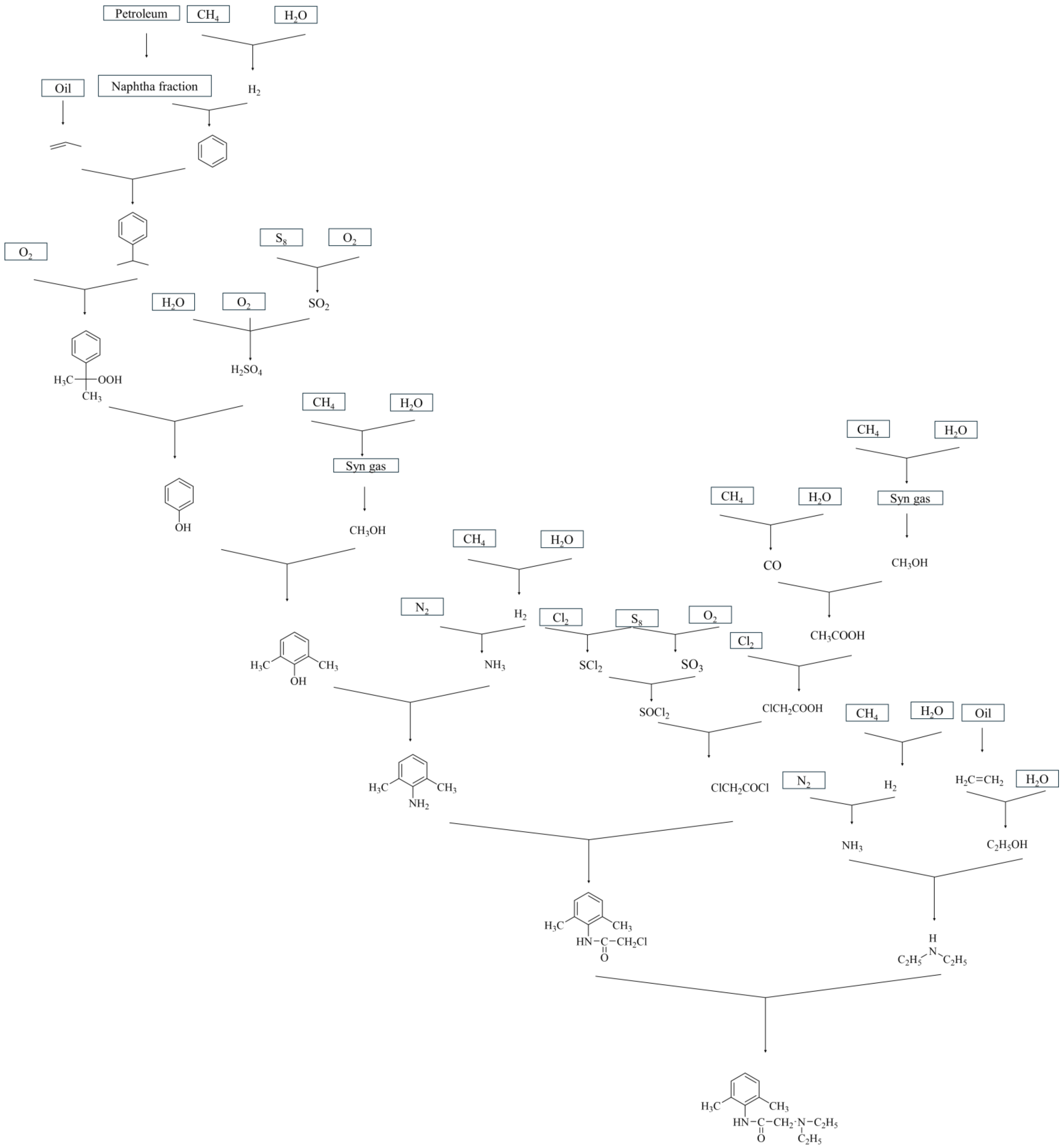

<u>Figure S2</u>: Synthesis tree of lidocaine[2,4]

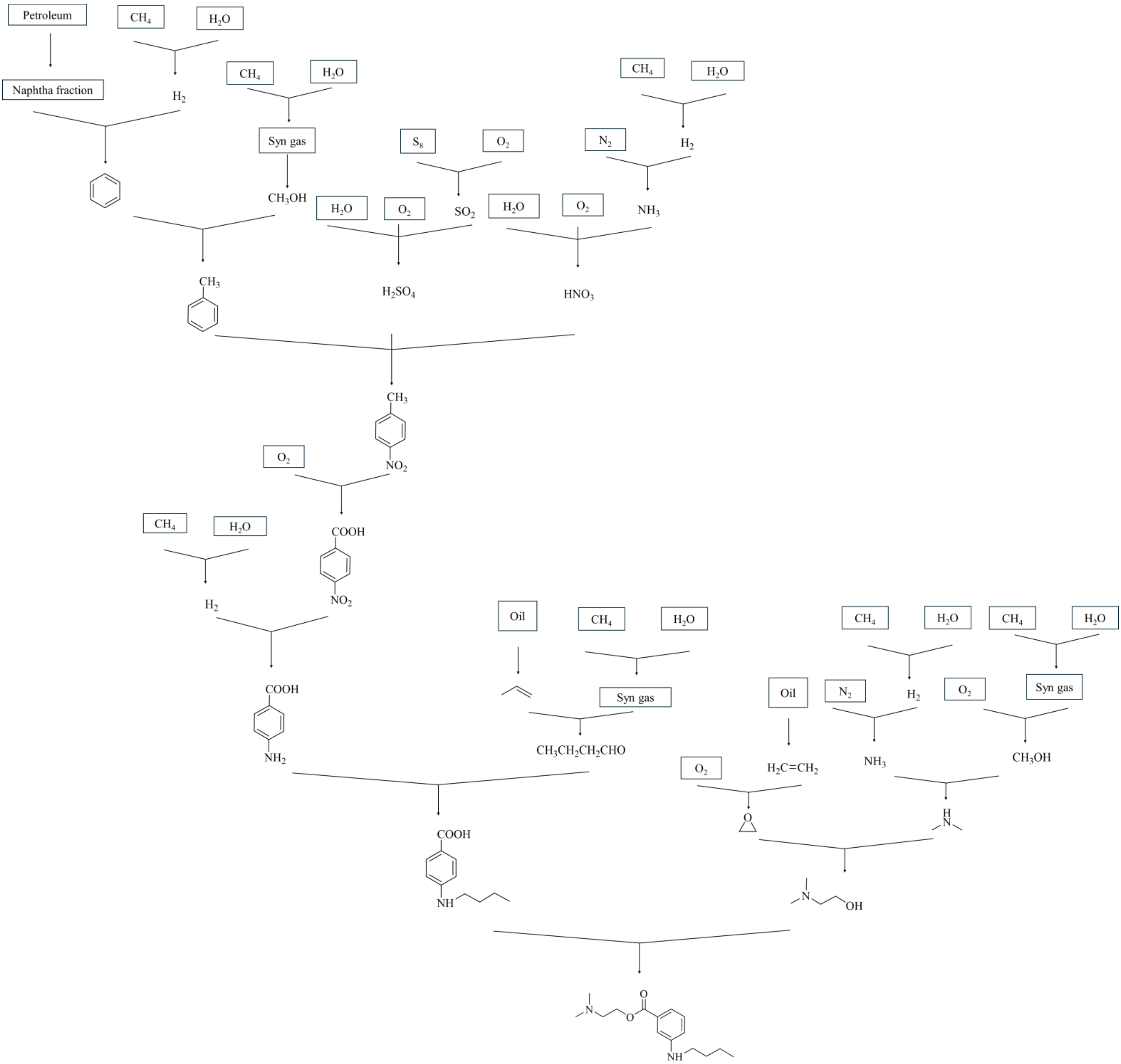

<u>Figure S3</u>: Synthesis tree of tetracaine [2,4]

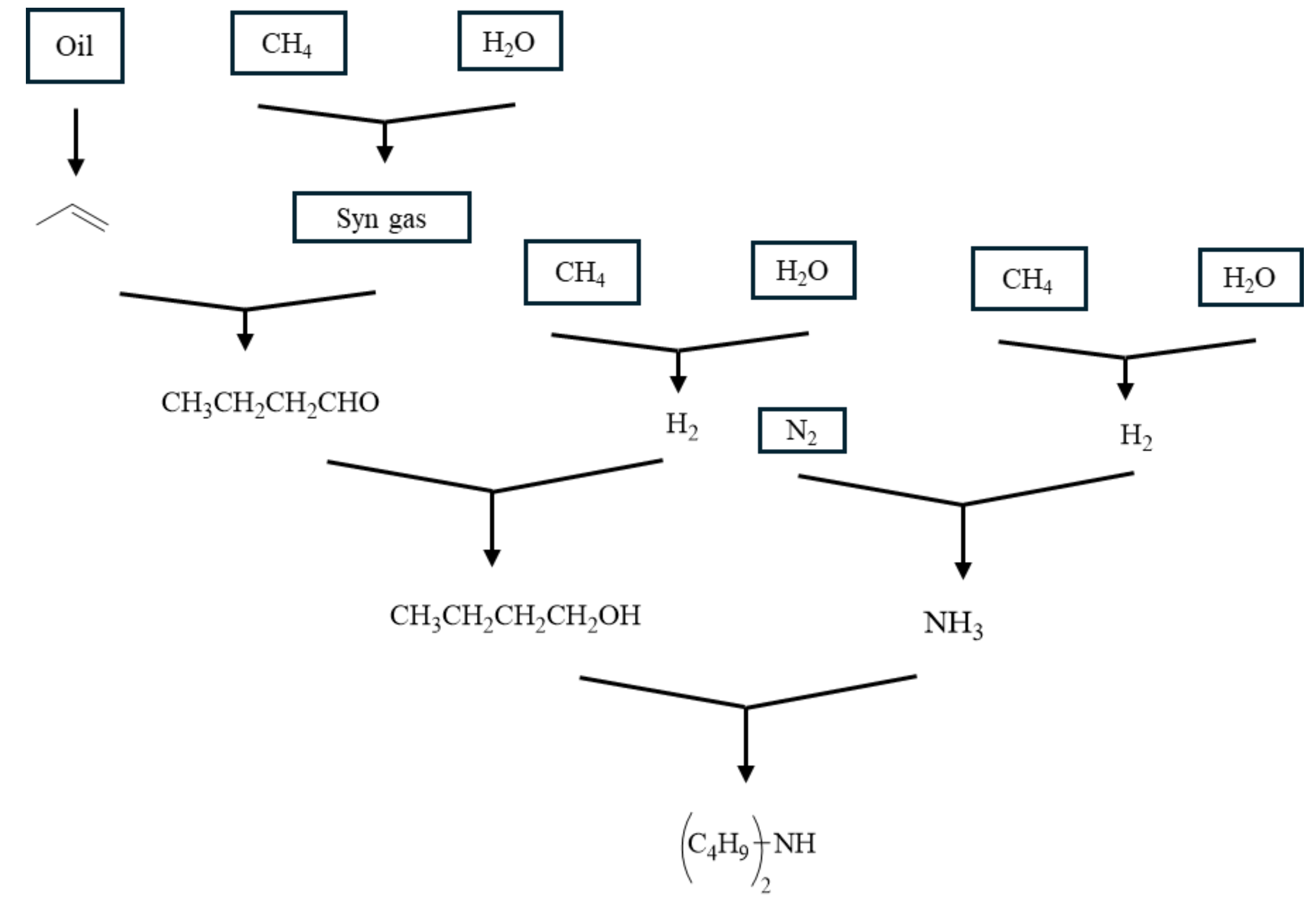

Figure S4: Synthesis tree of dibutylamine (DBA) [2,4]

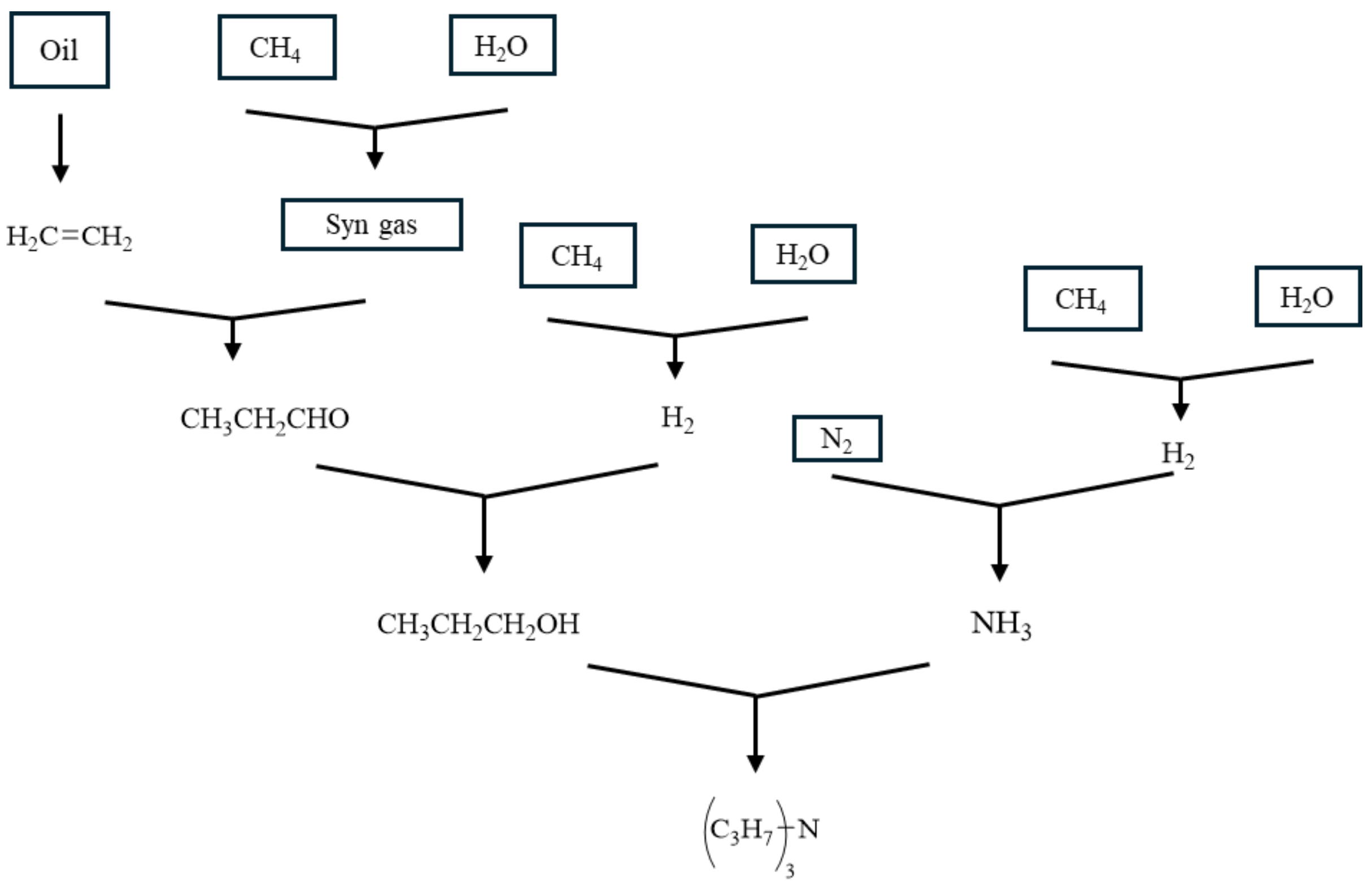

Figure S5: Synthesis tree of tripropylamine (TPA)[2,4]

References

(1) Sangster, J. Octanol Water Partition Coefficients of Simple Organic Compounds. *J. Phys. Chem. Ref. Data* **1989**, *18* (3), 1111–1229. https://doi.org/10.1063/1.555833.

(2) *National Center for Biotechnology Information. (n.d.). PubChem. https://pubchem.ncbi.nlm.nih.gov*.

(3) Strichartz, G. R.; Sanchez, V.; &chard Arthur, G.; Chafetz, R.; Martin, D. *Fundamental Properties of Local Anesthetics. 11. Measured Octanol:Buffer Partition Coefficients and PK, Values of Clinically Used Drugs*; 1990; Vol. 1.

(4) Jessop, P. G. Searching for Green Solvents. *Green Chemistry* **2011**, *13* (6), 1391–1398. https://doi.org/10.1039/c0gc00797h.

(5) Prat, D.; Wells, A.; Hayler, J.; Sneddon, H.; McElroy, C. R.; Abou-Shehada, S.; Dunn, P. J. CHEM21 Selection Guide of Classical- and Less Classical-Solvents. *Green Chemistry* **2015**, *18* (1), 288–296. https://doi.org/10.1039/c5gc01008j.
